# Inhaled and intravenous application of a stimulator of the soluble guanylate cyclase (BAY 41-8543) reduces pulmonary vascular resistance in a model of septic shock

**DOI:** 10.1186/1471-2210-11-S1-P41

**Published:** 2011-08-01

**Authors:** Nils Kronas, Birte Peters, Alwin E Goetz, Jens C Kubitz

**Affiliations:** 1 Department of Anesthesiology, University of Hamburg, Hamburg, Germany

## Background

Cardiac failure in septic shock usually originates from hypovolaemia, impaired global contractility or right ventricular failure (RVF). Acute RVF due to endotoxin-mediated pulmonary hypertension results in global hypoperfusion. However, the treatment of pulmonary vascular resistance (PVR) in septic patients remains a black box.

## Methods

After ethical approval, septic shock was induced in 32 pigs ( 25 ± 3 kg) by continuous infusion of endotoxin (Escherichia coli serotype 0111:B4). The animals received a protocol-based treatment with fluids and vasopressors according to the surviving sepsis campaign guidelines. Then, they were randomized to either inhaled (i.h.) (240μg kg^-1^) or intravenous (i.v.) (24μg kg^-1^) treatment with BAY 41-8543 or controls. Heart rate (HR (bpm)), mean arterial pressure (MAP (mmHg)), mean pulmonary artery pressure (MPAP (mmHg)), cardiac output (CO (l min^-1^)) and PVR (dyn sec cm^-5^) were assessed every 15 minutes for 1 h after starting the treatment. Following a wash out period of 1 h, the animals were once more randomized to double dose (D2) i.h. or i.v. respectively or to simultaneous administration of Bay 41-8543 at a single dose together with NO (i.h.) at 20 ppm. Hemodynamic measurements were taken for another hour. Data are expressed as mean ± SD at shock and 30 minutes after treatment. Differences between groups were analyzed using a linear mixed-effects model.

## Results

Both during i.h. and i.v. application of BAY 41-8543, a significant decrease in PVR and an increase in CO was observed. Additive inhaled NO decreased PVR more than doubling the dose of BAY 41-8543 (Table 1).

**Table 1:** 

	shock ***(n=32)***	i.h. ***(n=12)***	i.v. ***(n=14)***	con (n=6)	i.h. D2 (n=6)	i.h. no ***(n=5)***	i.v. D2 (n=5)	i.v. no (n=5)	con no (n=5)
**HR**	127 (34)	150 (21)	163** (28)	123 (34)	140 (35)	146 (19)	171 (29)	148 (29)	113 (34)
**MAP**	58 (7)	71 (7)	68 (6)	73 (8)	75 (6)	69 (9)	68 (14)	73 (10)	73 (8)
**MPAP**	38 (7)	40 (7)	37 (8)	44 (6)	42 (10)	42 (9)	40 (16)	37** (9)	39** (9)
**PVR**	1246 (401)	491** (106)	469** (198)	972 (325)	691** (322)	573** (245)	849* (815)	460** (103)	887** (296)
**CO**	2.02 (0.51)	4.65** (0.76)	5.04** (1.43)	2.93 (0.89)	3.73 (1.71)	4.16* (1.44)	4.3** (2.85)	4.06** (0.78)	2.46 (0.67)

* p<0.05   **p<0.01   vs. control group (con)

## Conclusion

The stimulator of the soluble guanylate cyclase BAY 41-8543 offers a treatment option for pulmonary hypertension in septic shock. Both inhalative and intravenous administration of BAY 41-8543 reduces PVR and increase CO. Further, there seems to be an additive effect of inhaled NO.

